# Effect of SiO_2_ amount on heterogeneous base catalysis of SiO_2_@Mg–Al layered double hydroxide†

**DOI:** 10.1039/c8ra04925d

**Published:** 2018-08-06

**Authors:** Mahiro Shirotori, Shun Nishimura, Kohki Ebitani

**Affiliations:** School of Materials Science, Japan Advances Institute of Science and Technology 1-1 Asahidai Nomi Ishikawa 923-1292 Japan s_nishim@jaist.ac.jp +81-761-51-1149 +81-761-51-1613; Graduate School of Advanced Science and Technology, Japan Advances Institute of Science and Technology Japan

## Abstract

The effects of SiO_2_ amount on the base catalysis of highly active finely crystallized Mg–Al type layered double hydroxides prepared by the co-precipitation method with coexistence of SiO_2_ spheres, denoted as SiO_2_@LDHs, were investigated. With the Si/(Mg + Al) atomic ratios of 0–0.50, the highest activity for the Knoevenagel condensation was observed in the case of Si/(Mg + Al) = 0.17, as the reaction rate of 171.1 mmol g(cat)^−1^ h^−1^. The base activity increased concomitantly with decreasing LDH crystallite size up to Si/(Mg + Al) atomic ratio of 0.17. However, above the Si/(Mg + Al) atomic ratio of 0.17, the reaction rate and TOF_base_ were decreased although the total base amount was increased. Results of TEM-EDS and ^29^Si CP-MAS NMR suggest that the co-existing SiO_2_ causes advantages for dispersion and reduction of the LDH crystallite to improve the base catalysis of SiO_2_@Mg–Al LDH, whereas the excess SiO_2_ species unfortunately poisons the highly active sites on the finely crystallized LDH crystals above a Si/(Mg + Al) atomic ratio of 0.17. According to these results, we inferred that the amount of spherical SiO_2_ seeds in the co-precipitation method is an important factor to increase the base catalysis of SiO_2_@LDHs; *i.e.* the control of Si/(Mg + Al) atomic ratio is necessary to avoid the poisoning of highly active base sites on the LDH crystal.

## Abstract 2

The effects of SiO_2_ amount on the base catalysis of highly active finely crystallized Mg–Al LDH(s) prepared by the co-precipitation method with coexistence of SiO_2_ sphere seeds were investigated by XRD, ICP, ^29^Si NMR, TEM-EDS and other techniques.

## Graphical abstract

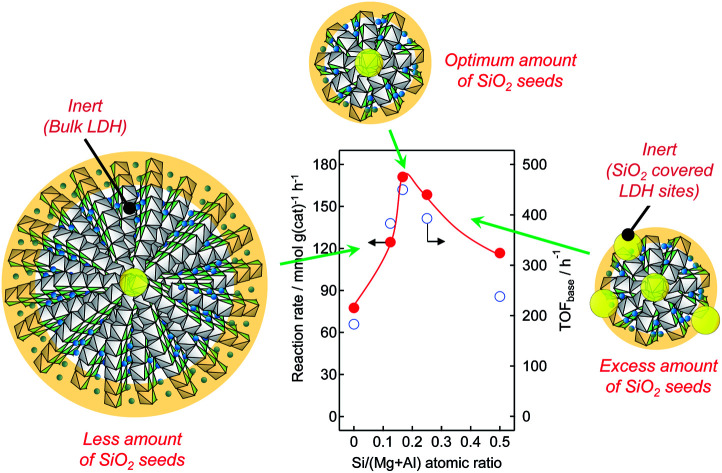

## Introduction

Heterogeneous solid catalysts have been widely used in various chemical reactions in the chemical industry, exhaust gas purification, and environmentally friendly reactions. They are more suitable for reactions at high temperatures than homogeneous catalysts, and are removed easily from the reactor. In particular, solid acid catalysts are used in many important processes related to petroleum refining and petrochemical production. Consequently, numerous studies have specifically examined solid acid catalysts. On the other hand, fewer efforts have been devoted to heterogeneous base catalysts.^1^ Because general base sites on a solid base catalyst are readily poisoned by moisture and carbon dioxide in the atmosphere,^2^ the development of a promotion strategy for solid base catalysts is more difficult than for solid acid catalysts.

Layered double hydroxide, generally called LDH, is a well-known layered clay mineral that is known to act as a unique solid base catalyst. Actually, LDH comprises brucite-like positively charged two-dimensional sheets denoted as [M_1−*x*_^2+^M_*x*_^3+^(OH)_2_]^*x*+^ and interlayer parts denoted as A_*x*/*n*_^*n*−^·*m*H_2_O, where A^*n*−^ corresponds to interlayer anions such as carbonate and hydroxide. The positively charged sheets and the interlayers are alternately laminated to compensate for the charge between sheets.^3–5^ Base sites on LDH are mainly regarded as identical Brønsted basic OH^−^ and HCO_3_^−^ anions, which are adsorbed onto the LDH surface. These unique basic sites can act even in an air atmosphere and can exhibit basic characteristics without pretreatment.^4^ As a solid base catalyst, LDH is well-known to catalyze various organic transformations such as aldol condensation,^6–9^ Knoevenagel condensation,^10–13^ epoxidation,^14–16^ and transesterification.^17–19^ Recent studies have indicated that LDHs can promote advanced environmentally friendly reactions such as biomass-derived saccharide conversion^13,20–25^ and photocatalytic conversion of CO_2_ in an aqueous solution.^26–28^ Therefore, the development of highly active LDH catalysts is eagerly sought.

The main base sites on the LDH surface are generally regarded as adsorbed anions located at the corner and edge of a crystal.^29^ However, the anions in the interlayer space cannot participate in the chemical reactions because of the high charge density of the LDH layers and the high contents of anionic species and water molecules, resulting in strong interlayer electrostatic interactions between the sheets.^4,30^ Therefore, the delamination of LDH nanosheets^30–36^ and the fine crystallization of LDH on appropriate carriers^37–42^ have been conducted to increase the number of exposed active base sites. As-prepared LDH materials also have been evaluated carefully to assess their characteristics and utility as photocatalysts and electrocatalysts,^43,44^ high active base catalysts for Knoevenagel condensation^45^ and epoxidation,^46^ magnetic separation of proteins,^37^ pseudocapacitance,^38^ flame retardancy of epoxy resins,^40^ and as adjuvants.^41^ Nevertheless, no report describes a study of improvement of base catalysis of LDH itself by fine crystallization followed by *in situ* growth method of SiO_2_@LDH nanoparticles.

An earlier study revealed a co-precipitation method for preparation of small-crystallized LDH catalysts on SiO_2_ nanospheres and explored the superior base catalyses for the Knoevenagel condensation of benzaldehyde compared with conventional LDHs.^47^ This method is applicable for the preparation of SiO_2_@LDH nanoparticles with various compositions and element combinations: *i.e.* SiO_2_@M^2+^–M^3+^LDH (M^2+^: Mg^2+^ or Ni^2+^, M^3+^: Al^3+^ or Ga^3+^, and M^2+^/M^3+^: 1 or 3). Various characterizations of SiO_2_@LDH nanoparticles using XRD, TEM-EDS, and ^29^Si CP-MAS NMR techniques revealed that the co-existence of small SiO_2_ sphere (*ca.* 40 nm diameter) surface generated the starting points of LDH growth *via* Si–O–M covalent bond formation, leading to the formation of fine-crystallized LDH and enhancement of base catalysis for the Knoevenagel condensation of benzaldehyde with ethyl cyanoacetate. However, the roles of Si–O–M covalent bonds in the fine-crystallization of LDH and base catalysis have not been explored well. Therefore, in this paper, we investigated the base properties and structural parameters of as-prepared SiO_2_@Mg–Al LDH materials with Mg^2+^/Al^3+^ = 3, and discussed the base catalysis with different Si to (Mg + Al) ratios to reveal the mechanism of our strategy.

## Experimental

### Materials and synthesis of catalysts

Tetraethyl orthosilicate (TEOS), triethanolamine (TEA), and benzaldehyde were purchased from Sigma-Aldrich Corp. Sodium carbonate (Na_2_CO_3_), sodium hydroxide (NaOH) and toluene were supplied by Kanto Chemical Co. Inc. Cetyltrimethylammonium bromide (CTAB), magnesium nitrate hexahydrate (Mg(NO_3_)_2_·6H_2_O), aluminum nitrate enneahydrate (Al(NO_3_)_3_·9H_2_O), benzaldehyde and benzoic acid were obtained from Wako Pure Chemical Industries Ltd. Benzaldehyde was purified by distillation under 0.4 Pa pressure. Ethyl cyanoacetate was purchased from Tokyo Chemical Industry Co. Ltd. and was used without further purification.

Spherical SiO_2_ (40 nm) was prepared according to descriptions in earlier reports.^41,46^ First, 96 mmol of TEA and 2 mL of TEOS were combined in a 200 mL eggplant flask. The two-phase mixture was heated in an oil bath at 363 K for 20 min without stirring. When the mixture was removed from the oil bath, 26.0 mL of an aqueous solution (2.8 wt%) of CTAB pre-heated at 333 K was added immediately as a structure-directing agent in a condensation process. Then, it was stirred continuously for 24 h at room temperature. Thereafter, the resulting mixture was added to 50 mL of ethanol to obtain colloidal aqueous suspension. The obtained precipitate was centrifuged for 5 min at 4000 rpm. After decantation, the sediment was re-dispersed through vigorous stirring in 50 mL of an ethanolic solution of ammonium nitrate (20 g L^−1^), and then refluxed for 1 h. This procedure was repeated three times. The same operation was performed with a solution of concentrated hydrochloric acid in ethanol (5 g L^−1^) to replace the ammonium ions. The final sediment was washed with ethanol, and then dried *in vacuo*. The obtained spherical SiO_2_ powder was calcined at 823 K under 1 L min^−1^ of air flow for 6 h.

The SiO_2_@(*Z*)LDH catalysts (*Z*: desired Si/(Mg + Al) atomic ratio) were prepared *via* an *in situ* co-precipitation method according to a previous report.^42^ Spherical SiO_2_ (40 nm) was dispersed in 20 mL of water using ultrasound treatment. After 30 min, 0.96 mmol of Na_2_CO_3_ was added to the solution. Then, after an additional 5 min of sonication was conducted, 19.2 mL of metal nitrate aqueous solution ([Mg] + [Al] = 0.075 M) was slowly dropped into the spherical SiO_2_ dispersed solution, followed by stirring at room temperature. The pH was maintained at 10.0 by an aqueous NaOH solution (1 M) during titration. The obtained suspension was stirred for an additional 1 h. After the resulting paste was filtered, it was washed with 1 L of water and ethanol. Then, it was dried at 383 K overnight. The Si/(Mg^2+^ + Al^3+^) atomic ratios were varied from 0 to 0.50 whereas the Mg/Al atomic ratio was adjusted to 3.

### Reaction

Knoevenagel condensation of benzaldehyde with ethyl cyanoacetate was proceeded in a 20 mL Schlenk tube under an N_2_ flow (30 mL min^−1^). The reaction was performed using 1.0 mmol of benzaldehyde, 1.2 mmol of ethyl cyanoacetate, 10 mg of catalysts and 3 mL of toluene at 313 K. The obtained products were analyzed using a GC-FID (GC-2014, Shimadzu Corp.) equipped with a polar column (DB-FFAP, Agilent Technologies Inc.).

### Characterizations

X-ray diffraction patterns (XRD) were collected using a SmartLab (Rigaku Corp.) with a Cu Kα X-ray source (40 kV, 30 mA). The LDH (003) and (110) crystallite sizes were calculated using the Scherrer equation: *D*_*hkl*_ = *Kλ*/(*β* cos *θ*) (*K*: Scherrer number (0.9), *λ*: incident ray wavelength (0.1542 nm), *β*: peak width at half height (rad), *θ*; Bragg angle). ^29^Si cross polarization magic angle spinning nuclear magnetic resonance (^29^Si CP-MAS NMR) measurements were obtained by an Avance III 500 (Bruker Analytik GmbH) in a 4 mm ZrO_2_ rotor. The spinning rate was 8 kHz. The ^29^Si chemical shifts are referenced to hexamethylcyclotrisiloxane (taken to be at *δ* = −9.6875 ppm). Transmission electron microscope – energy dispersive X-ray spectroscopy (TEM-EDS) elemental mapping analytical techniques were done with a JEM-ARM200F (JEOL) at 200 kV. Inductively coupled plasma – atomic emission spectrometry (ICP-AES) was operated by an iCAP 6300 Duo (Thermo Fisher Scientific Inc.) to estimate the actual amount of precipitated M(OH)_*x*_ and SiO_2_ in as-prepared SiO_2_@Mg–Al LDHs with various Si/(Mg + Al) atomic ratio.

## Results and discussion

### Optimization of Si/(Mg + Al) ratio in SiO_2_@LDH

We prepared SiO_2_@LDHs with various loading amounts of SiO_2_ to ascertain the optimized SiO_2_@LDH structure for high catalytic reactivity. The sphere morphology and the diameter of SiO_2_ were confirmed from SEM and TEM observations, as presented in an earlier report.^47^ The correlation between Si/(Mg + Al) atomic ratio, base catalysis, and structural properties of SiO_2_@LDHs were investigated in the range of 0–0.50 on Si/(Mg + Al) atomic ratio. The prepared catalysts are designated as SiO_2_@(*Z*)LDH, where *Z* is a Si/(Mg + Al) atomic ratio. The actual ratios of Si/(M^2+^ + M^3+^) and M^2+^/M^3+^ in the obtained materials are presented in Table 1.

**Table 1:** Chemical compositions of as-prepared SiO_2_@(*Z*)LDHs estimated by ICP-AES

Sample	Si/(M^2+^ + M^3+^) ratio		M^2+^/M^3+^ ratio	
	Precursor	Obtained material	Precursor	Obtained material
LDH(CP)			3.0	2.8
SiO_2_@(0.13)LDH	0.13	0.14	3.0	2.5
SiO_2_@(0.17)LDH	0.17	0.18	3.0	2.6
SiO_2_@(0.25)LDH	0.25	0.28	3.0	2.5
SiO_2_@(0.50)LDH	0.50	0.53	3.0	2.2

**Fig. 1:**
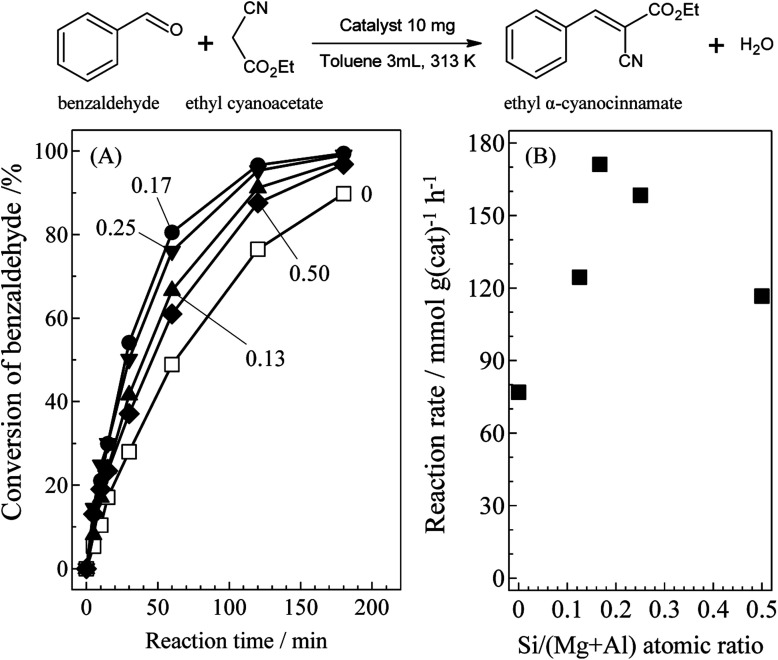
Activities for Knoevenagel condensation of benzaldehyde with ethyl cyanoacetate over as-prepared SiO_2_@LDHs with various Si/(Mg + Al) atomic ratio; (A) time-based reaction progression on benzaldehyde conversion and (B) reaction rate. Reaction conditions: benzaldehyde (1.0 mmol), ethyl cyanoacetate (1.2 mmol), catalyst (10 mg), toluene (3 mL), 313 K, N_2_ flow (30 mL min^−1^). Number in (A) denotes Si/(Mg + Al) atomic ratio.

Fig. 1 presents catalytic activity for the Knoevenagel condensation over SiO_2_@(*Z*)LDHs as (A) time-based reaction progression on benzaldehyde conversion and (B) reaction rate. The detailed results are listed in Table S1 (see ESI†). Among various Si/(Mg + Al) atomic ratio from 0 to 0.50, actually, the 0.17 was found to be the best catalyst with a reaction rate of 171.1 mmol g(cat)^−1^ h^−1^. This reaction rate is 2.2 times higher than conventional LDH prepared with the same co-precipitation method without SiO_2_ seeds (Si/(Mg + Al) = 0).

**Fig. 2:**
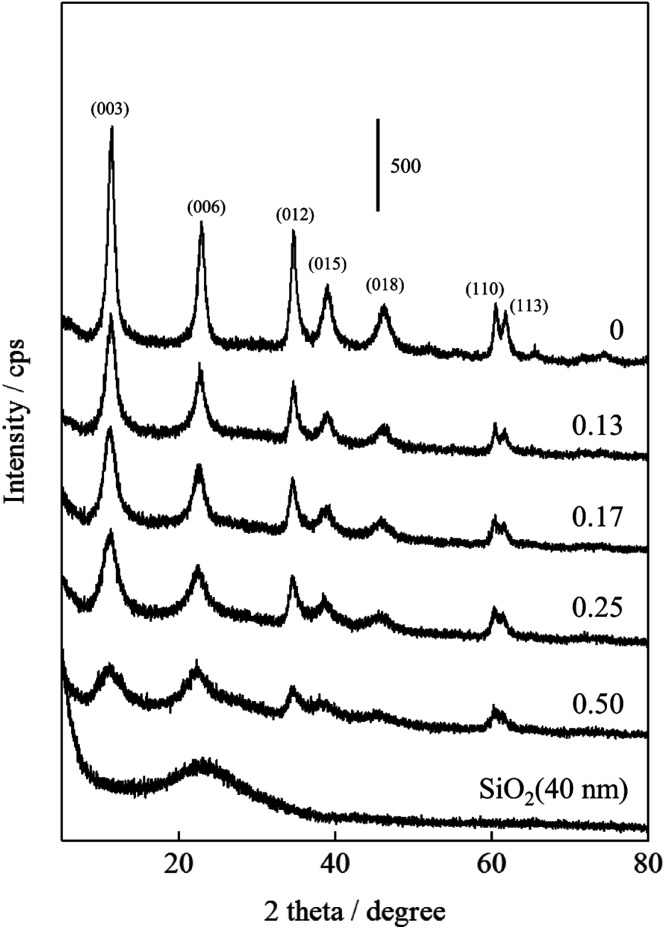
XRD patterns of as-prepared SiO_2_@LDHs with various Si/(Mg + Al) atomic ratio.

**Fig. 3:**
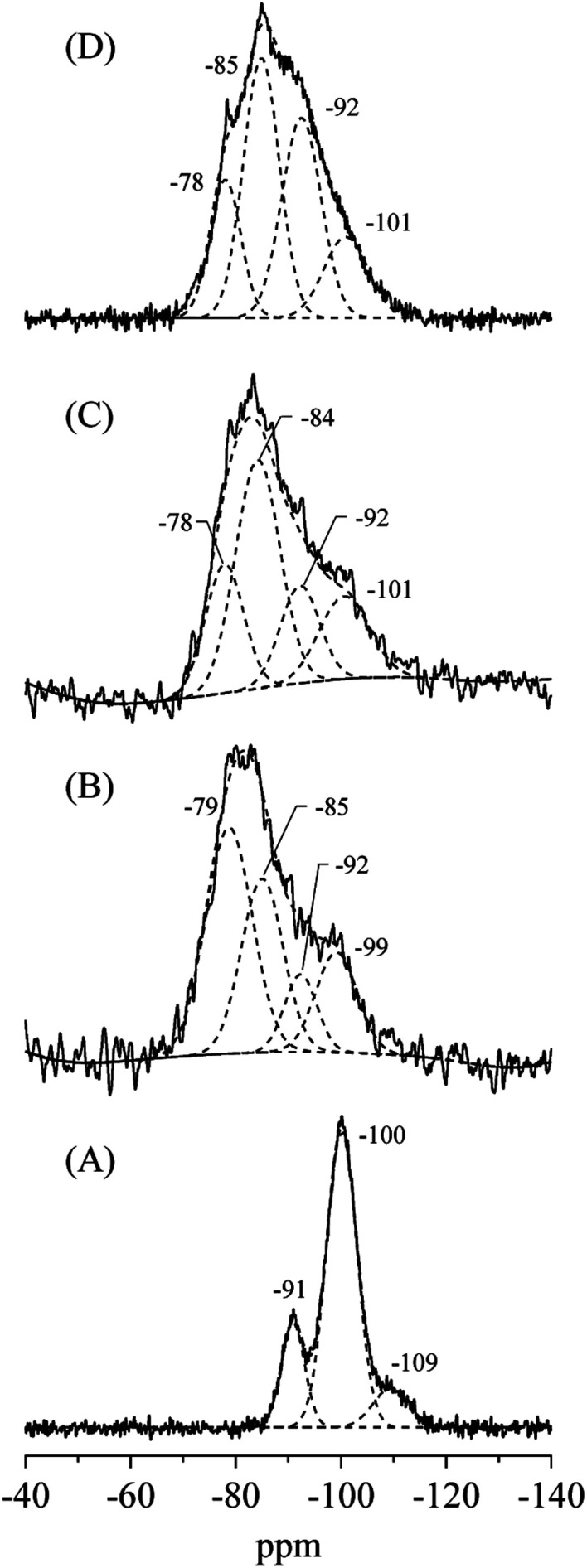
^29^Si CP-MAS NMR spectra of (A) spherical SiO_2_ (40 nm), (B) SiO_2_@(0.13)LDH, (C) SiO_2_@(0.17)LDH and (D) SiO_2_@(0.50)LHD.

The XRD patterns and crystal properties of SiO_2_@(*Z*)LDHs are portrayed respectively in Fig. 2 and Table 2. All prepared catalysts showed an LDH-originated diffraction pattern. The intensity of LDH originated peaks decreased in accordance with Si loading amount, whereas that of amorphous SiO_2_ increased slightly. Lattice parameters *a* and *c*, respectively calculated from LDH (003) and (110) diffraction peaks, are almost identical among Si/(Mg + Al) atomic ratios of 0–0.50. This result indicates clearly that these SiO_2_@(*Z*)LDHs have the same LDH crystal unit. However, the crystallite size of LDH is unquestionably affected by Si/(Mg + Al) atomic ratio. The crystallite size of *D*(003) is reduced in accordance with the Si loading amount. The crystallite size of *D*(110) is almost identical in the region among Si/(Mg + Al) ratio of 0–0.13, but it is reduced from *ca.* 13 nm to 8 nm when a Si/(Mg + Al) ratio increased. In our earlier research, it was revealed that the co-precipitation method with co-existence of spherical SiO_2_ caused dispersion of starting points of LDH crystal growth on the SiO_2_ surface through the Si–O–Al and Si–O–Mg covalent bonds to lead generation of fine-crystallized LDH nanocrystal.^47^Fig. 3(A) shows that the spherical SiO_2_ (40 nm) showed three peaks at −91, −100 and −109 ppm, which respectively correspond to Q^2^, Q^3^, and Q^4^ species^48–50^ where Q^*n*^ designated the Si-centered tetrahedral structural species; Q refers to silicon atom and *n* denotes the number of bridging oxygens. Furthermore, SiO_2_@(*Z*)LDHs showed broad resonance between −70 to −115 ppm, which include some peaks attributed to Q^0^ and/or Q^1^ (−60 to −83 ppm)^49^ and a Si-centered tetrahedral structure that possesses Si–O–Al and Si–O–Mg bonds (−73 to −105 ppm).^42^

**Table 2:** Crystal properties of as-prepared SiO_2_@LDHs with various Si/(Mg + Al) atomic ratio

Si/(Mg + Al) atomic ratio	Lattice parameter *c*/nm	Crystallite size (003)^a^/nm	Lattice parameter *a*/nm	Crystallite size (110)^a^/nm
0	2.33	7.6	0.31	13.4
0.13	2.32	5.2	0.31	13.7
0.17	2.36	4.5	0.31	10.3
0.25	2.38	3.7	0.31	8.0
0.50	2.43	2.8	0.31	8.3

a The crystallite sizes of LDHs were calculated by Scherrer equation: *D*_*hkl*_ = *Kλ*/(*β* cos *θ*) (*K*: Scherrer number (0.9), *λ*: incident ray wavelength (0.1542 nm), *β*: peak width at half height (rad)).

These results suggest that, in the case of lower Si/(Mg + Al) atomic ratio (<0.13), the LDH crystal is immobilized onto the SiO_2_ surface through the Si–O–Al and Si–O–Mg bonds to inhibit *ab*-face stacking without reducing the plane crystallite size. This result seems to be attributable to the lower number of starting points of LDH crystal growth: the amount of metal constituting one crystal did not change compared with conventional LDH prepared without SiO_2_ seeds (Si/(Mg + Al) = 0). Actually, the proportion for the ^29^Si CP-MAS NMR peaks attributed to Si–O–Mg and Si–O–Al bonds on the SiO_2_@(0.13)LDH is only ≤40%, whereas that of SiO_2_@(0.17)LDH is ≤61%, as shown in Fig. 3 and Table 3. Therefore, we infer that the number of Si–O–Mg and Si–O–Al bonds on the SiO_2_ surface deeply affected reduction of the crystallite size not only in the stacking direction but also in the plane direction when below Si/(Mg + Al) < 0.17–0.25.

**Table 3:** Surface area and silicon environments in the SiO_2_ and SiO_2_@(*Z*)LDHs as determined by ^29^Si CP-MAS NMR

Sample	Assignment	*δ*/ppm	Percentage/%
SiO_2_ (40 nm) sphere	Q^4^	−109	11
	Q^3^	−100	70
	Q^2^	−91	19
SiO_2_@(0.13)LDH	Q^3^	−99	17
	Q^2^	−92	10
	Q^4^(3Al), Q^3^(1Mg)		
	Q^4^(4Al), Q^3^(1Al), Q^2^(1Mg)	−85	30
	Q^1^ and/or Q^0^	−79	43
SiO_2_@(0.17)LDH	Q^3^	−100	18
	Q^2^	−92	17
	Q^4^(3Al), Q^3^(1Mg)		
	Q^4^(4Al), Q^3^(1Al), Q^2^(1Mg)	−85	44
	Q^1^ and/or Q^0^	−78	21
SiO_2_@(0.50)LDH	Q^3^	−101	14
	Q^2^	−92	31
	Q^4^(3Al), Q^3^(1Mg)		
	Q^4^(4Al), Q^3^(1Al), Q^2^(1Mg)	−85	37
	Q^1^ and/or Q^0^	−78	17

Correlation between the base amount and catalytic activity for Knoevenagel condensation over SiO_2_@(*Z*)LDHs is presented in Table 4. Although the base amount of SiO_2_@(0.13)LDH was lower than that of conventional LDH, the reaction rate and the apparent TOF per base site (TOF_base_) for SiO_2_@(0.13)LDH are higher than those of LDH. The *D*(003) of SiO_2_@(0.13)LDH was smaller than LDH, whereas *D*(110) of SiO_2_@(0.13)LDH and LDH are almost identical, as shown in Table 2. Therefore, these indicated that the immobilization of LDH crystal onto SiO_2_ with inhibition of the *ab*-face stacking led to increase in the number of highly active base sites located on the surface LDH layer. Above the Si/(Mg + Al) atomic ratio of 0.13, a base amount increased in accordance with Si/(Mg + Al) ratio from 0.32 to 0.49 mmol g(cat)^−1^. Furthermore, the activity was maximized at Si/(Mg + Al) ratio of 0.17 with the reaction rate of 171.1 mmol g(cat)^−1^ h^−1^. It is particularly interesting that the reaction rate per obtained LDH phase and TOF_base_ were also maximized at Si/(Mg + Al) ratio of 0.17 with the reaction rate of 193.6 mmol g(LDH)^−1^ h^−1^ and TOF_base_ of 450 h^−1^. Above the Si/(Mg + Al) ratio of 0.17, both the reaction rate per LDH phase and TOF_base_ were decreased respectively to 158.4 mmol g(LDH)^−1^ h^−1^ and 238 h^−1^ at Si/(Mg + Al) ratio of 0.50. These results strongly suggest that the Si/(Mg + Al) atomic ratio affects not only the LDH crystallite size and base amount but also the type of base sites and these fractions.^51^

**Table 4:** Activity for the Knoevenagel condensation and base amount of as-prepared SiO_2_@(*Z*)LDHs

Sample	Amount of LDH^a^/wt%	Reaction rate^b^		Base amount^c^/mmol g(cat)^−1^	TOF_base_/h^−1^
		mmol g(cat)^−1^ h^−1^	mmol g(LDH)^−1^ h^−1^		
LDH(CP)	100	76.9	76.9	0.42	183
SiO_2_@(0.13)LDH	90.8	124.5	137.0	0.32	389
SiO_2_@(0.17)LDH	88.3	171.1	193.6	0.38	450
SiO_2_@(0.25)LDH	82.9	158.4	191.0	0.40	396
SiO_2_@(0.50)LDH	73.6	116.7	158.4	0.49	238

a The amount of LDH in the SiO_2_@(*Z*)LDHs was calculated by ICP-AES with an assumption: all SiO_2_@(*Z*)LDHs are composed of mixture of LDH and SiO_2_.

b Reaction rate for the Knoevenagel condensation of benzaldehyde with ethyl cyanoacetate.

c Base amount calculated from poisoning test by benzoic acid titration.

The LDH crystallite size of SiO_2_@(0.50)LDH is at least smaller than that of SiO_2_@(0.17)LDH. Therefore, the base catalysis of SiO_2_@(0.50)LDH is expected to be better than that of SiO_2_@(0.17)LDH if the base catalysis is only influenced by the crystallite size. ^29^Si CP-MAS NMR spectra showed that the proportion of terminal Si–OH species assigned as Q^0^ and/or Q^1^ decreased in accordance with Si/(Mg + Al) atomic ratio, as shown in Table 3, indicating first that a surface Si–O–Si bond is cleaved to generate terminal Si–OH species and then that these act as cross-link point with Mg and Al ions. Consequently, when there are the excess free terminal Si–OH species in the solution after the generation of SiO_2_@LDH, these excess Si–OH species cover the LDH crystal to produce Si–O–Mg and Si–O–Al covalent bonds. Although the base amount is increased even the region from SiO_2_@(0.13)LDH to SiO_2_@(0.50)LDH, this phenomenon might take place only with difficulty on the inferior base sites located at a flat plane of LDH. However, the decrease of TOF_base_ strongly suggests that the high active base sites are poisoned by Si species in the case of a higher Si/(Mg + Al) atomic ratio.

**Fig. 4:**
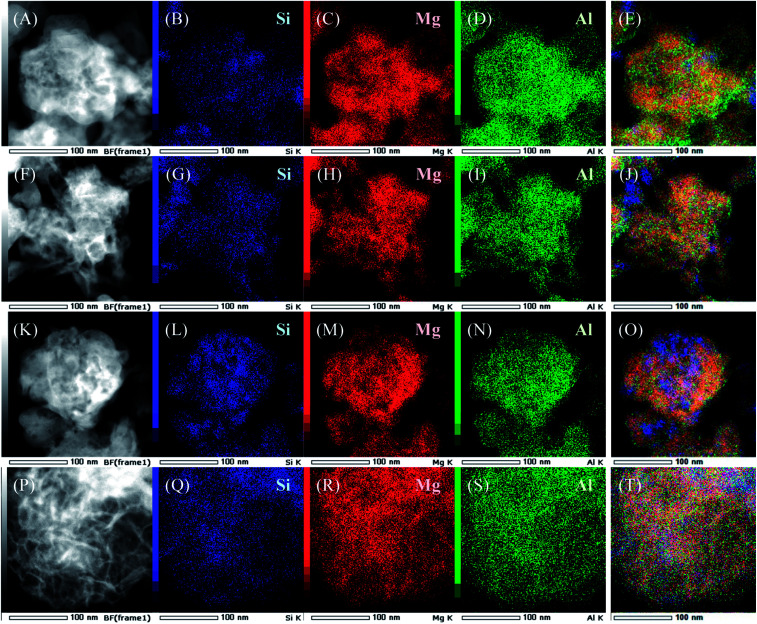
(A, F, K and P) Dark-field TEM images of as-prepared SiO_2_@LDHs with various Si/(Mg + Al) atomic ratio: (A) 0.13, (F) 0.17, (K) 0.25 and (P) 0.50. Also shown are (B–E), (G–J), (L–O), (Q–T) EDS mapping results of as-prepared SiO_2_@LDH with various Si/(Mg + Al) atomic ratio.

Dark-field TEM images and results of EDS elemental mapping of SiO_2_@(*Z*)LDHs are presented in Fig. 4. In the case of lower Si/(Mg + Al) atomic ratio such as 0.13 and 0.17, the LDH crystal is generated with covering the SiO_2_ phase to form a SiO_2_ core – LDH shell-like structure, as shown in Fig. 4(A)–(J). Furthermore, results show that the boundary between SiO_2_ phase and LDH phase becomes ambiguous in accordance with the increase of Si/(Mg + Al) atomic ratio (Fig. 4(K)–(T)). These indicate that first the LDH crystal grows up from the SiO_2_ surface to generate the immobilized SiO_2_ core – LDH shell structure. If there are excess dissolved SiO_2_ species possessing a free terminal Si–OH group, then these produced Si–O–Mg and Si–O–Al covalent bonds with the LDH crystal to cover the LDH shell.

Accordingly, we infer the correlation between Si/(Mg + Al) atomic ratio and base catalysis of prepared SiO_2_@(*Z*)LDHs as follows: (i) below Si/(Mg + Al) atomic ratio of 0.13, the LDH crystal is just immobilized onto the SiO_2_ surface with inhibition of the *ab*-face stacking. The exposed corner and edge located at the surface layer act as highly active base sites. (ii) Fine crystallization occurs not only in the stacking direction, but also in the plane direction to increase the amount of base sites, especially base sites with high activity up to Si/(Mg + Al) atomic ratio of 0.17. (iii) Above a Si/(Mg + Al) atomic ratio of 0.17, excess terminal Si–OH species covered the highly active base sites to produce Si–O–Mg and Si–O–Al covalent bonds, thereby lowering the activity.

## Conclusions

The effects of SiO_2_ loading amounts on the crystallite sizes, basicity, and catalytic activity of SiO_2_@LDH catalysts were investigated. SiO_2_@LDHs were prepared using co-precipitation with the coexistence of various amounts of spherical SiO_2_ with particle sizes of *ca.* 40 nm. The XRD results suggest that the LDH crystallite size of *D*(003) is simply reduced in accordance with the Si loading amount. Furthermore, the crystallite size of *D*(110) is almost identical below Si/(Mg + Al) of 0.13, although it is reduced above 0.13. Base catalysis of SiO_2_@LDHs was evaluated using Knoevenagel condensation of benzaldehyde and ethyl cyanoacetate. Both the reaction rate and the apparent TOF per base site were increased in the region between Si/(Mg + Al) atomic ratio of 0–0.17, whereas the base amount is increased linearly from the Si/(Mg + Al) atomic ratio of 0.13 to 0.50 with reduction of the LDH crystallite size. The results of ^29^Si CP-MAS NMR and STEM-EDS suggest that a surface Si–O–Si bond is cleaved, generating terminal Si–OH species that act as a cross-link point with Mg and Al ions to form an immobilized SiO_2_ core – LDH shell structure. However, when the amount of Si becomes excessive with respect to Mg and Al ions, the excess Si–OH group forms Si–O–Mg and Si–O–Al covalent bonds with LDH crystal to cover the LDH shell. From these results, we inferred the effect of SiO_2_ amount on heterogeneous base catalysis of SiO_2_@Mg–Al LDH as follows: (i) below Si/(Mg + Al) atomic ratio of 0.13, the highly active base sites located at the corner and edge of surface layer are exposed by immobilization of LDH crystals onto the SiO_2_ surface, (ii) up to Si/(Mg + Al) atomic ratio of 0.17, the number of exposed highly active base sites is increased in accordance with reduction of LDH crystallite, and (iii) above Si/(Mg + Al) atomic ratio of 0.17. Excess terminal Si–OH species covered the highly active base sites through the Si–O–Mg and Si–O–Al covalent bonds to decrease the activity. This study elucidated the correlation between SiO_2_ amount, crystal properties, and the basicity of fine-crystallized SiO_2_@LDH catalysts to present a new technique to improve the base catalysis of the widely used LDH material. After optimization of the Si/(Mg + Al) atomic ratio in SiO_2_@LDHs, the Si/(Mg + Al) atomic ratio of 0.17 was found to be the best catalyst, with the reaction rate of 171.1 mmol g(cat)^−1^ h^−1^, which is a 2.2 times higher value than that of the conventional LDH prepared with same protocol in absence of SiO_2_ seed agents.

## Conflicts of interest

There are no conflicts to declare.

## Supplementary Material

RA-008-C8RA04925D-s001
