# Supplementary material for: Effect of SiO2 amount on heterogeneous base catalysis of SiO2@Mg–Al layered double hydroxide
Source: RSC Adv. 2018 Aug 6;8(49):28024–31. doi: 10.1039/c8ra04925d (PMC9083954; doi:10.1039/c8ra04925d)
Supplement: RA-008-C8RA04925D-s001 [file RA-008-C8RA04925D-s001.pdf]

Electronic Supplementary Material (ESI) for RSC Advances.
This journal is © The Royal Society of Chemistry 2018

Supporting Information for

Effect of SiO_2 amount on Heterogeneous Base Catalysis of SiO_2@Mg-Al Layered Double Hydroxide

Mahiro Shirotori,[a] Shun Nishimura [a,b,*] and Kohki Ebitani [a,b,*]

[a] School of Materials Science, Japan Advances Institute of Science and Technology, 1-1 Asahidai, Ishikawa, Nomi, 923-1292, Japan

[b] Graduate School of Advanced Science and Technology, Japan Advances Institute of Science and Technology

Contact Author

S.N.: Tel. +81-761-51-1613; Fax. +81-761-51-1149; e-mail, s_nishim@jaist.ac.jp.

Table S1 Reaction progress based on time for Knoevenagel condensation of benzaldehyde with ethyl cyanoacetate over as-prepared SiO_2@LDHs with various Si/(Mg+Al) atomic ratio.

Si/(Mg+Al) /molecular ratio	Time /min	Conversion of BA /%	Conversion of EC /%	Yield (based on BA) /%	Carbon balance /%
0	5	5.4	1.9	6.4	102.2
	10	10.4	6.3	10.5	101.4
	15	17.1	12.4	14.9	99.0
	30	28.0	20.3	25	98.8
	60	48.9	39.0	46.8	99.1
	120	76.5	61.8	79.8	104.4
	180	89.8	73.6	85.8	97.5
0.13	5	8.2	4.6	11.8	104.2
	10	17.1	11.8	17.8	101.7
	15	23.9	17.4	24.0	101.1
	30	41.6	31.8	40.6	97.5
	60	66.6	51.9	66.4	101.0
	120	91.2	71.8	94.7	104.5
	180	97.7	77.4	101.1	104.3
0.17	5	13.0	10.7	14.6	101.7
	10	21.1	17.4	22.3	101.3
	15	29.9	24.6	29.1	99.6
	30	54.1	44.3	54.5	101.3
	60	80.5	66.5	81.3	101.6
	120	96.6	80.1	96.5	100.7
	180	99.4	82.7	97.7	99.1
0.25	5	14.4	11.7	14.7	100.6
	10	24.8	20.3	22.8	98.6
	15	30.0	24.3	27.3	98.1
	30	50.1	40.6	49.1	100.1
	60	76.0	63.1	75.3	100.1
	120	95.3	80.0	97.3	102.5
	180	99.0	83.2	103.6	104.8
0.50	5	13.1	8.4	12.0	100.1
	10	19.0	13.8	17.4	99.5
	15	23.4	17.5	21.4	99.1
	30	37.1	29.7	32.8	96.7
	60	61.0	49.4	57.0	97.1
	120	87.6	70.9	92.3	105.6
	180	96.8	79.6	93.4	97.7

Reaction conditions: benzaldehyde (BA, 1.0 mmol), ethyl cyanoacetate (EC, 1.2 mmol), catalyst (10 mg), toluene (3 mL), 313 K, N_2 flow (30 mL min^{-1}).
